# On the Diseases of Infants and Children

**Published:** 1856-12

**Authors:** 


*On the Diseases of Infants aud Children.* By FLEETWOOD CHURCHILL, M.D. M.R.I.A. Hon. Fellow of the College of Physicians, Ireland. Second American Edition, Enlarged and Revised by the Author. Edited, with Additions, by WM. V. KEATING, M.D. A.M. Physician to St. Joseph's Hospital, Lecturer on Obstetrics, &c. Philadelphia, Blanchard & Lea, 1856.

The former edition of Dr. Churchill's work on Diseases of Children, has been before the profession several years, and its merits are consequently well known. The present edition has been carefully revised by the author, and copious references made to American writers on the several topics discussed, the object being to adapt it especially to the American market.

The present volume contains seven hundred and thirty-five pages, and is divided into two parts.

Part I. is "On the Management of Infancy and Childhood," and embraces Chapters on the following subjects, viz.:—

1. Preliminary Observations on the Management of Infancy and Childhood. 2. Management of the Infant at Birth. 3. The Food of Infancy and Childhood. 4. Cleanliness. 5. Air and Exercise.

Part II. is on "The Diseases of Infancy and Childhood," and contains Chapters on the following topics:—

1. Intra-Uterine or Congenital Diseases. 2. Cephalœmatoma—Fractures of the Cranium. 3. Irritation of the Nervous System—Trismus Nascentium. 4. Chorea—St. Vitus' Dance. 5. Convulsions. 6. Acute Meningitis—Acute Hydrocephalus. 7. Chronic Hydrocephalus. 8. Inflammation of the Brain. 9. Tumors of the Brain. 10. Conjestion and Apoplexy of the Brain. 11. Paralysis. 12. Intra-Uterine Diseases of the Respiratory Organs. 13. Spasm of the Glottis—Thymic Asthma. 14. Hooping Cough. 15. Croup. 16. Atelectasis Pulmonum. 17. Bronchitis. 18. Pneumonia. 19. Pleurisy. 20. Phthisis. 21. Malformation of the Heart. 22. Pericarditis. 23. Endocarditis. 24. Congenital Malformation of the Digestive Organs. 25. Dentition. 26. Inflammation of the Mouth. 27. Apthœ and Thrush. 28. Miguet, or Pseudo-Membranous Inflammation. 29. Ulcerated Sore Mouth. 30.

Gangrene of the Mouth, or Cancerum Oris. 31. Tonsilitis. 32. Parotitis. 33. Pseudo-Membranous Pharyngitis. 34. Putrid Sore Throat. 35. Post Pharyngeral Abscess. 36. Diseases of the Stomach. 37. Indigestion or Weaning Brash. 38. Gastritis. 39. Diarrhœa. 40. Dysentery. 41. Intestinal Worms. 42. Jaundice and Enlargement of the Liver and Spleen. 43. Tabes Mesenterica. 44. Peritonitis. 45. Diseases of the Skin—under this head, the author treats of the usual list of Chronic Cutaneous Affections and the Eruptive Fevers. 46. Infantile Remittent Fever. 47. Typhoid Fever. 48. Infantile Syphilis.

From the enumeration of the contents of the work, our readers will see that it is a comprehensive treatise, embracing almost everything relating to the diseases of infancy and early childhood, whether congenital or non-congenital. The method pursued by Dr. Churchill, in writing his work, is strictly that of a *compiler*, rather than of an original thinker and experienced practitioner. And while we cannot recommend the work to the exclusion of the valuable treatises by Stewart, Condie, and Meigs of our own country, we can cordially assure our readers that they will find it a very valuable addition to their libraries.

For sale by D. B. Cooke & Co. Lake Street, Chicago.

